# Monitoring Immune Responses to Vaccination: A Focus on Single-Cell Analysis and Associated Challenges

**DOI:** 10.3390/vaccines13040420

**Published:** 2025-04-16

**Authors:** LaToya Montgomery, Anis Larbi

**Affiliations:** 1 Medical and Scientific Affairs, Beckman Coulter Life Sciences, Brea, CA 92821, USA; lmontgomery02@beckman.com; 2 Department of Medicine, Faculty of Medicine and Health Sciences, University of Sherbrooke, Sherbrooke, QC J1K 2R1, Canada

**Keywords:** immune response, vaccination, flow cytometry, reproducibility

## Abstract

Monitoring the immune response to vaccination encompasses both significant challenges and promising opportunities for scientific advancement. The primary challenge lies in the inherent complexity and interindividual variability of immune responses, influenced by factors including age, genetic background, and prior immunological history. This variability necessitates the development of sophisticated, highly sensitive assays capable of accurately quantifying immune parameters such as antibody titers, T-cell responses, and cytokine profiles. Furthermore, the temporal dynamics of the immune response require comprehensive longitudinal studies to elucidate the durability and quality of vaccine-induced immunity. Challenges of this magnitude pave the way for immunological research advancements and diagnostic methodologies. Cutting-edge monitoring techniques, such as high-throughput sequencing and advanced flow cytometry, enable deeper insights into the mechanistic underpinnings of vaccine efficacy and contribute to the iterative design of more effective vaccines. Additionally, the integration of analytical tools holds the potential to predict immune responses and tailor personalized vaccination strategies. This will be addressed in this review to provide insight for enhancing public health outcomes and fortifying preparedness against future infectious disease threats.

## 1. Introduction

The critical role of vaccine research in preventing infectious diseases, reducing morbidity and mortality, and promoting global health cannot be overstated. One of the first prevention strategies, the inoculation of the smallpox virus, was performed in the 18th century in Constantinople and reported by Lady Mary Montagu [1]. While this has faced resistance for various reasons (religious, medical, economic, and even sexism), this incident was apparently the first clinical trial (tested on prisoners and orphans) to open the field of prophylaxis for infectious diseases. Yet, a few decades later, Edward Jenner used cowpox instead of smallpox to mitigate the risks; thus, leading to the emergence of the vaccination from those attempts (the Latin word for cow is *vacca*). Since then, vaccination has played a pivotal role in eradicating smallpox, but also in drastically reducing the prevalence of diseases such as polio and measles and controlling outbreaks of influenza as well as other infectious agents. Not only do reports indicate vaccine efficacy in disease prevention, but they also depict the substantial economic benefits of vaccination programs. Vaccination campaigns have direct and indirect impacts on the economy of low-/middle-/high-income countries. This is nicely summarized [2] and further demonstrated for more recently developed vaccines, such as the COVID-19 vaccine and 15- and 20-valent pneumococcal conjugate vaccines [3,4]. However, sustained support for vaccine research remains a cornerstone of public health initiatives aimed at protecting global populations from infectious diseases and reducing the burden on public healthcare systems.

A vaccination clinical trial typically follows a structured and multi-phase workflow to ensure both safety and efficacy [5]. The process begins with conducting preclinical research, incorporating laboratory experiments and animal studies, and gathering initial safety and immune-response data. Upon successful preclinical results, the trial moves into clinical phases (Phase 1 and Phase 2). While Phase 1 involves a small group of healthy volunteers to assess safety and determine dosage levels [6], its success is pivotal for progression to Phase 2, which includes a larger group of participants for vaccine immunogenicity evaluations and further safety assessments [7]. In Phase 3, the trial is expanded to thousands of participants across multiple locations to statistically confirm the vaccine’s relevance and monitor any possible side effects. Throughout all phases, data are meticulously collected, analyzed, and reviewed by regulatory bodies. If the vaccine meets all safety and efficacy criteria, it is submitted for approval by relevant health authorities. Post-approval, Phase 4 trials may be conducted to monitor long-term effects, ensure ongoing safety in the general population, or develop further hypotheses on the mechanism of action (immune response) leading to optimal responses. Each of these steps requires rigorous data quality to guarantee that vaccines are both safe and effective before being made publicly available. Improving the reproducibility of clinical research ultimately helps to deepen our understanding of the immune response and is necessary to reach clinical trial generalizability within different settings (such as ethnicity, age, gender, and environment).

## 2. The Immune Response

**Figure 1:**
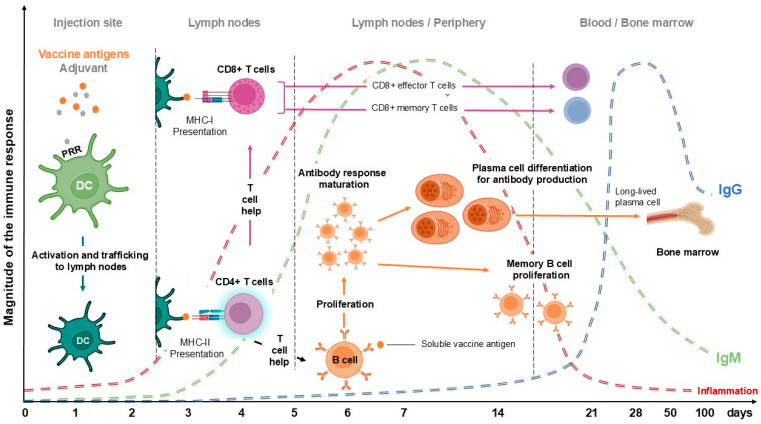
**The temporality of the immune response following vaccination.** Following vaccination, the antigen-presenting cells migrate to lymph nodes and initiate the immune response, including inflammation leading to activation of the corresponding T cells, which in turn help B cells to produce antibodies (IgM and then IgG). DC: dendritic cells; PRR: pattern recognition receptor; Ig: immunoglobin.

The kinetics of the immune response to vaccination are characterized by a precise and coordinated sequence of events involving both the innate and adaptive immune systems (Figure 1). Upon vaccine administration, the innate immune response is rapidly triggered, within minutes to hours, through pattern recognition receptors (PRRs), which are activated on dendritic cells and macrophages [8]. These antigen-presenting cells then migrate to the lymph nodes, where they present processed antigens to naïve T and B lymphocytes, thereby initiating the adaptive immune response [9]. Approximately seven to 10 days post-vaccination, a primary wave of antigen-specific effector T and B cells are triggered, marked by specific antibody plasma cells [10]. The peak of this primary antibody response typically occurs around two to four weeks post-vaccination, followed by a contraction phase with most effector cells undergoing apoptosis [11], leaving behind a reservoir of memory cells (Figure 1). These memory B and T cells persist long-term and can be rapidly reactivated upon subsequent exposure to the antigen, providing a more robust and rapid secondary immune response. The longevity and magnitude of the memory response can vary based on factors such as the nature of the antigen, the type of vaccine, the adjuvants used, and the host’s immunological history [12]. Understanding these temporal dynamics is crucial for optimizing vaccine schedules and enhancing the efficacy of immunization programs.

A multitude of factors tend to be influential and play a crucial role in determining the efficiency and durability of the induced immunity. One of the primary factors is the nature of the antigen used in the vaccine, which can significantly affect the type and magnitude of the immune response. For instance, protein subunit vaccines often require adjuvants to heighten immunogenicity by promoting antigen uptake and presentation [13]. The choice of adjuvant itself is another critical factor due to its capacity for modulating various innate immune pathways and steering the subsequent adaptive response [14]. Additionally, the route of vaccine administration (e.g., intramuscular, subcutaneous, or mucosal) can impact the localization and immune cell-type activation to direct the immune response [15]. Host factors such as age, genetic background, and pre-existing immunity also play significant roles. For example, older individuals may exhibit immunosenescence, which may result in a weakened vaccine efficacy [16]. Pre-existing immunity from past infections or previous vaccinations can lead to enhanced responses through memory cell activation and diminished responses because of immune exhaustion, interference, or even unwanted health issues [17].

During the immune response, a myriad of soluble factors play an essential role by acting as key components during immunity development or inflammation, which is the collective term for the process. When a vaccine is administered, the body recognizes vaccine antigens as foreign substances, which triggers an innate immune response characterized by the activation of various immune cells such as macrophages, dendritic cells, and neutrophils [18] that release pro-inflammatory cytokines, including interleukins (IL-1, IL-6) and tumor necrosis factor-alpha (TNF-α), which contribute to the localized inflammatory response at the site of injection [19]. This inflammation helps to recruit additional immune cells to the site and promotes the maturation and migration of dendritic cells to lymph nodes (Figure 1), where they present antigens to T and B cells to initiate the adaptive immune response. The controlled inflammatory response is important for the development of a robust and long-lasting immunity, as it facilitates the formation of memory T and B cells that can quickly respond to future exposures to the pathogen [20]. However, excessive or prolonged inflammation can lead to adverse effects and is a topic of ongoing research for optimizing vaccine formulations and adjuvants that enhance immune responses while minimizing potential side effects [21]. Overall, inflammation is a critical aspect of the immune response to vaccination, playing a pivotal role in the efficacy and safety of vaccines.

## 3. Assessing the Response to Vaccination

**Figure 2:**
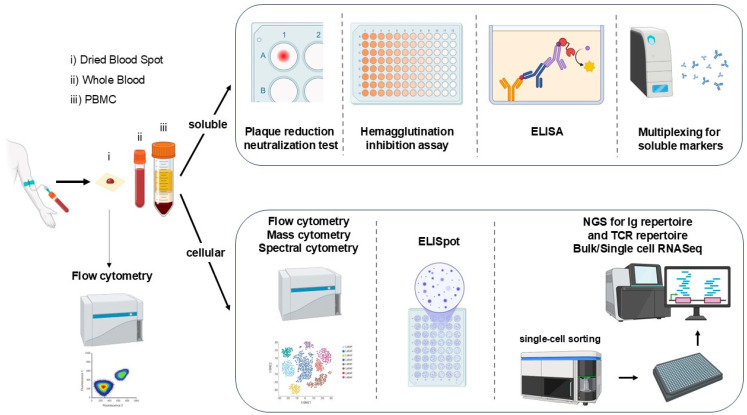
**Technologies used to monitor responses to vaccination.** Following blood sampling, analysis of whole blood or isolation of PBMC is performed. The plaque reduction neutralization test is used to quantify the titer of neutralizing antibodies for a virus. The serum is diluted and mixed with a viral suspension to allow the present antibodies to react with the virus. The effect of antibodies on the hemagglutination process, by which a virus binds to red blood cells, is measured with the hemagglutination inhibition assay rather than the titer needed to block the cytopathic effects of the virus. It is only a correlation of the ability of antibodies to inhibit virus infection of host cells. Analysis of soluble markers of the response (antibody levels, cytokines) can now be easily multiplexed with recent innovations. The cellular response is more often performed using high-dimension and single-cell analysis. Single-cell analysis can be performed with single-cell sorting before RNA sequencing (scRNA-seq). The analysis of immune cell composition from a dried blood spot has been shown to be possible by flow cytometry.

The first line of response assessed following vaccination is the ability of the organism to mount an antibody response. Hence, techniques such as quantitative enzyme-linked immunosorbent assays (ELISA) are used to assess the level of antibodies present. The hemagglutination inhibition test and the plaque reduction neutralization test (PRNT) are widely used to define the quality of antibodies produced. The hemagglutination inhibition test is more commonly used than PRNT, as it is simpler and does not require a virus culture facility [22]. PRNT, considered the gold standard for measuring neutralization antibodies, is based on the cytolytic activity of the virus, which forms plaques and foci in the cell culture. This method allows for the determination of a neutralization percentage, expressed as a foci neutralization capacity, using the virus-only control as the reference for the 100% foci formation. This is performed using blood samples collected before and several weeks after vaccine inoculation (Figure 2). While measuring the humoral response is useful to evaluate the efficacy of vaccines, only an analysis of the complex immune response can help identify how and why some individuals may not respond [23].

As the immune response is quite complex and follows a specific dynamic (Figure 1), the study of the cellular response to vaccines tends to be crucial for vaccine assessment and immunogenicity (Figure 2). Peripheral blood mononuclear cells (PBMCs) are commonly isolated from vaccinated individuals and stimulated with specific vaccine antigens to mimic in vivo exposures. Techniques such as enzyme-linked immunospot (ELISpot) assays can quantify antigen-specific T-cell responses by detecting cytokine production at the cellular level, providing detailed insights into the frequency and functionality of these cells [24]. Although the ELISpot assay was initially developed to detect antibody-secreting cells, it is widely used to evaluate IFN-γ production by T cells. It can be combined with intracellular cytokine staining to assess polyfunctional T cells [25]. Flow cytometry is the method of choice for immune monitoring, enabling the phenotypic and functional characterization of immune cells, including the expression of activation markers, intracellular cytokine staining, and proliferation assays [26]. Additionally, measuring cytokine levels in culture supernatants with multiplex bead assays or ELISA can provide a comprehensive profile of the inflammatory response induced by the vaccine antigens [27]. High-throughput sequencing techniques, such as RNA-seq, can further elucidate the transcriptomic changes in immune cells upon antigen stimulation, offering detailed molecular insights into the immune response [28]. These ex vivo assays are indispensable for understanding the quality and magnitude of immune responses elicited by vaccines, guiding the optimization of vaccine formulations and informing clinical development.

Whole blood measurement of immune response to vaccination offers a practical and comprehensive approach for assessing immunogenicity. This method involves the direct stimulation of whole blood samples with vaccine antigens, preserving the complex interplay between various cell types and soluble factors within the blood. One of the primary advantages of this approach is the minimal sample processing required, which reduces potential artifacts introduced by cell isolation procedures and maintains the physiological context of the immune response [29]. A procedure of this sort is particularly valuable in clinical settings with limited sample volume and allows for the simultaneous assessment of both cellular and humoral immune responses, making it a powerful tool for vaccine research and development [30].

Monitoring the vaccination immune response presents several significant hurdles, which complicates the evaluation of vaccine efficacy as well as the prediction of long-term protection. One major challenge is the inherent variability in immune responses among individuals, influenced by factors such as age, genetics, pre-existing immunity, and health status [31]. The complexity of the immune system necessitates the use of sophisticated technologies (high-dimensional flow cytometry, single-cell RNA sequencing, and multiplex assays) to capture a comprehensive picture of the immune response [32]. The standardization and validation of these assays across different laboratories also pose significant challenges for reproducibility [33]. Furthermore, the identification of reliable protection equivalents remains elusive for many vaccines, complicating the identification of the best predictive immune correlates. Logistic constraints, such as the need for longitudinal studies or the collection of multiple biological samples in different sites, serve as one final challenge adding to the complexity and cost of monitoring immune responses. One possibility to drastically reduce the logistic constraints is the utilization of dried blood spot material for immunological assessment of vaccine responses. Several reports demonstrate the feasibility of this approach with flow cytometry by measuring the composition of the immune system, serology, autoantibodies, inflammatory markers, genomic analysis, epigenetic profiling, and even metabolic features showing very good correlations with the serum-paired samples [34,35,36,37,38,39,40,41]. Various markers (CD3, CD4, CD8, CD14, CD16, CD19, CD45, CD56, and the corresponding subset proportions) were all preserved following one month of the dried blood spot storage. The ease of collection, storage, and shipping makes dried blood spot use an interesting possibility for large and/or multi-center studies as well as for regions with lower resources. While current regulations facilitate their use [42], further studies are needed to validate more markers of vaccination response, such as the analysis of antigen-specific T-cell persistence and function [43].

## 4. Other Assays Assessing the Immune Response

While post-vaccination assays assist with in vivo immune response assessment, other assays help with understanding key response elements and mechanisms. These data tend to be very useful during the pre-clinical stage of vaccine formulation. Goss-Dolin et al. [44] demonstrated the utility of using models such as whole blood assays to measure multiple immune cells, cytokine release activations, and monocyte migration assessment, as well as DC maturation and polarization T-cell abilities. In addition, DC/T cell interface assays help with antigen presentation evaluation and serve as validation for adjuvanticity and tolerability in mouse pre-clinical models. This entire phenomenon dictates the magnitude in which human immunity in vitro studies can provide mechanistic insights and help with novel immunomodulatory agents and vaccine developments. However, one limitation of the in vitro approach is the lack of spatial information pertaining to the immune response. While two-dimensional assays, such as whole blood and cell cultures, offer relative simplicity, they fail to replicate the complexity of the in vivo immune environment.

In contrast, three-dimensional assays, including organoids and organ-on-a-chip systems, can model tissue structures more accurately and closely mimic the in vivo setting with minimal ethical concerns. Nevertheless, these technologies fail to replicate the tissue morphology and provide low throughput. At the same time, these advanced assays require specialized skills and equipment, making them more complex to implement. Despite their complexity, three-dimensional assays hold significant potential for providing deeper perceptions into the spatial and functional immune response dynamics [45]. In recent years, spatial imaging methods have emerged for visualizing cell-to-cell interactions within tissues [46]. Spatial biology has propelled various research fields forward, including the immuno-oncology discipline. The demand for novel biomarkers to predict treatment responses has been a significant driver of technological advancements in this area. Gaining insights into the tumor microenvironment has also been crucial for understanding cancer evolution as well as immune cell types and their process interactions following treatment [47]. It is now possible to measure protein and genomic features that facilitate understanding of cellular distribution, cellular spatial relationships, and tissue heterogeneity. This is one of the objectives of the Human Atlas Project [48]; applying this to the injection site following vaccination may provide insightful information.

Proteomic data are not the only type of information that spatial imaging allows. In fact, transcriptomic data can also be generated by combining spatial and co-indexing of transcriptomes and epitopes (CITE)-seq methods. This modality is feasible for using antibody-derived DNA tags (ADTs) to stain a tissue slide and determine in-tissue barcoding of both DNA tags and mRNAs for spatially resolved high-plex protein and transcriptome co-profiling [49]. Each ADT contains a poly(A) tail, a unique molecular identifier, and a specific DNA sequence that is distinct to the corresponding antibody. One research study provides detailed technical information regarding the process [50]. With this method, Liu et al. demonstrated the ability to extract genomic information at the single-cell level in various tissue biopsies (tonsil, skin, thymus, and spleen). For this methodology, testing occurred at the injection site following SARS-CoV-2 mRNA vaccination, employing a total of 273 proteins with whole transcriptome analysis. Findings revealed that APCs and T cells were localized in spatially distinct regions, whereas B cells were distributed throughout the tissue. Moreover, a subset of T cells expressing activation genes (LAG3) associated with peripheral helper T cells (Tph) was identified and noted for local T-cell activation involvement in response to vaccination [51]. This again highlights the diversity of assays available to deepen our understanding of the immune response following vaccination.

## 5. Advanced Techniques for Immune Analysis

Optimizing the immune response to vaccination is possible when the various mechanism phases are understood. To deepen comprehension of cellular interactions and their role in the response, knowledge pertaining to individual cell behavior from both a proteomic and genomic angle is helpful. Therefore, only the main technologies driving the advances in single-cell analysis are described below (Figure 2 and Table 1): basically, flow cytometry and single-cell RNA sequencing (scRNA-seq).

Flow cytometry is a pivotal technology in immunological research and clinical diagnostics, enabling high-throughput multiparametric individual cell analysis within heterogeneous populations [52]. Utilizing the principles of hydrodynamic focusing, cells are aligned in a single stream within a sheath fluid and interrogated individually by one or more laser beams. The resulting forward scatter (FSC) and side scatter (SSC) are indicative of cell size and complexity, respectively. Concurrently, cells are labeled with fluorochrome-conjugated antibodies or probes that bind to specific surface or intracellular targets. The emitted fluorescence from these fluorochromes is amplified, detected, and converted into electronic signals for downstream analysis. Compensation algorithms between fluorochromes are applied for spectral overlap correction, which tends to be a critical step in multicolor flow cytometry [53]. Advanced techniques, such as spectral flow cytometry, capture the full emission spectrum, advancing the resolution and enabling the simultaneous analysis of up to 50 distinct parameters [54,55]. Likewise, innovations in high-dimensional data analysis and machine learning algorithms facilitate the interpretation of complex datasets, making flow cytometry an indispensable tool for immunophenotyping, functional assays, rare cell detection, and even small particle analysis [56,57]. These advancements continue to drive the field forward, providing deeper insights into cellular heterogeneity and immune responses.

The need for multiparametric assays originates from the scientific quest, encompassing responses to vaccination as well as natural infections. For instance, early discoveries show the need for a better understanding of the markers expressed in T cells, their relationship to B cell activity, and antibody production following vaccination [58]. This knowledge has enabled the identification of the superior capacity of polyfunctional T-cell subsets in comparison to T cells producing single cytokines in response to infectious agents [59]. Another example leveraging high-dimensional flow cytometry involves the profiling of yellow fever T-cell responses, which revealed the presence of polyfunctional T cells years after vaccination [60]. The study of the immunological memory to vaccines and their relationship to B cells is well covered and summarized [61]. More recently, flow cytometry analysis of the immune response to the mRNA SARS-CoV-2 vaccine revealed the maintenance of functional antigen-specific T cells six months after infection [62]. On the other hand, Sokal et al. used single-cell and repertoire profiling of the B-cell response to demonstrate that an antigen-driven activation persisted and matured up to six months after SARS-CoV-2 infection and may provide long-term protection [63].

There has been more and more interest in correlating immune profile protection in the context of natural infection and vaccination, specifically booster vaccination [64,65]. This is of particular interest for fragile groups such as older individuals (≥65 years) who may not develop a sufficient magnitude and/or persistence of immune protection [66]. While it was noted that booster vaccination had no significant effect on the T-cell compartment, the weaker B-cell primary response in older adults was significantly enhanced, reaching levels comparable to those observed in younger adults. Again, these data suggest the necessity for better understanding of the immune correlates of protection to help support future vaccine research and development in applicable areas, including human papillomavirus (HPV) [67], malaria [68], yellow fever [69], human immunodeficiency virus (HIV) [70], tuberculosis [71] and many other vaccine areas.

One study portrayed vaccines comprising antigenic peptides conjugated to a glycolipid agonist (glycolipid-peptide [GLP] vaccine) generated more efficient long-lived CD8^+^ liver-resident memory T cells, which are crucial for protection against the liver-stage of infection [68]. The CD8^+^ T cells were primed by a combination of type 1 conventional dendritic cells and inflammatory signals and boosted in a natural killer T cell-independent manner. This aspect regarding the organ-specific effect of vaccination is important and may benefit from recent technological developments within the field of spatial biology. While understanding the localized response is important, knowing the immunological history before vaccination is also relevant. As described earlier [72], protection against influenza correlated with polyfunctional CD4^+^ and CD8^+^ T cells and subsets of helper T cells (T-follicular helper and Thelper type 17), while increased susceptibility was attributed to pro-inflammatory populations such as γδ T cells, CD16^neg^ NK cells, and CD8^+^ T cells producing only TNFα. The use of advanced flow cytometers offering a larger array of parameters could help decipher the complexity of the immune response [73]. This is what spectral flow cytometry and mass cytometry can offer. Unlike traditional flow cytometry, which uses specific bandpass filters to detect signals from fluorochromes, spectral flow cytometry captures the full emission spectrum of each fluorochrome over a wide range of wavelengths [74]. Many studies have shown the potential of such a technique in the context of vaccination, and while this may be seen as partly exploratory, it provides novel information, such as (i) the association of CD31+ naïve Tcells to immunosenescence and response to vaccinations in older individuals [75], (ii) better understanding of B-cell transition to memory B cells following vaccination [76], (iii) the potential role of γδ T cells in recently developed HIV-1 vaccines [77], (iv) how to assess germinal characteristics in response to SARS-CoV-2 vaccination [78], and many more [79]. Another high-dimensional cytometry approach is CyTOF (cytometry by time of flight), a mass cytometry technique with little signal overlap. CyTOF uses antibodies conjugated to rare heavy metal isotopes instead of fluorescence. The mass cytometer detects the unique time-of-flight of each metal with detection overlaps limited to <2% [80]. This significantly reduces the spectral overlap that needs to be corrected with compensation when using fluorescent antibodies. Moreover, CyTOF exhibits a lower background, which improves the detection of low-expression markers. This approach has been used in the context of vaccination [81,82]. One significant advantage of mass cytometry is the identification of the specificity of the T cells being analyzed. The combination of mass cytometry with major histocompatibility complex tetramers has enabled the characterization of epitopes recognized by T cells [83]. Due to variable staining intensity and spectral overlap, fluorescence cytometry is limited compared to mass cytometry [84]. Mass cytometry has even enabled the identification of neo-antigen-specific CD8+ T cells [85] and opens the door to further discoveries. A comparative analysis of single-cell methodologies is provided in Table 1.

Single-cell RNA sequencing (scRNA-seq) has revolutionized vaccine immune response monitoring by offering detailed insights into cellular heterogeneity and dynamic gene expression profiles. This technique measures transcriptomes at the single-cell level, enabling their identification and functional characterization [86]. Additionally, scRNA-seq facilitates the discovery of novel cell types, states, and developmental trajectories, providing crucial insights into immune activation, differentiation, and memory formation. This detailed understanding aids in pinpointing biomarkers and gene expressions linked to effective vaccine responses or adverse effects, leading to better vaccine designs and personalized immunization strategies [87,88,89]. Generally, heterogeneous samples are stained with a series of antibodies to identify the desired subset and isolated by single-cell sorting in individual wells [90], which are then submitted for sequencing. By combining single-cell sorting for VDJ and RNA sequencing, Sheid et al. characterized B-cell responses against SARS-CoV-2 [91]. The SARS-CoV-2-specific B-cell repertoire consisted of transcriptionally distinct B-cell populations with cells producing potently neutralizing antibodies (memory and activated B cells). Another example by Kotliarov et al. used scRNA-seq to analyze the magnitude baseline features affecting the immune response to influenza and yellow fever [92]. The authors identified baseline blood transcriptional signatures that predicted antibody responses to influenza and yellow fever vaccinations in healthy individuals. By using CITE-seq to profile surface proteins and transcriptomes for over 50,000 single cells from both high and low influenza vaccination responders, they discovered that these baseline signatures indicated activation levels in a plasmacytoid dendritic cell/B lymphocyte network. Each of these examples underscores the power of the scRNA-seq in uncovering the cellular and molecular mechanisms underlying vaccine-induced immunity. This knowledge paves the way for refined vaccine design and personalized immunization strategies.

## 6. Data Integration, Analysis, and Future Perspectives

The importance of data integration and analysis has become increasingly relevant within a multitude of disciplines, including cancer biology, infectious diseases, and immunological research. This is particularly true when various characteristics and parameters such as cellular, epigenomic, transcriptomic, proteomic, and microbiome profiles are included in the same study [93] to find a mechanistic explanation of observed phenomena. Whether the scope of data integration pertains to immune response, vaccination, or an alternate entity, one major challenge relates to devising a harmonized approach to ensure that data are structured, robust, and of high quality. To facilitate data integration and analysis, it is essential to have a strategic approach to ensure that the research or trials are properly structured for yielding high-quality, reliable, and meaningful data [94]. Particularly with flow cytometry, experiments or trials should be designed to generate high-quality data by minimizing potential sources of variability. Implementing the right controls, ample sample collection and processing, sufficient marker subset identification, and single-cohort subject division for repeat experimentation, pending adequate sample and staining controls, are a few strategic measures for high-quality designing [95]. One of the most fundamental steps during cytometry experiment planning involves ensuring acceptable statistical power with appropriate sample sizes and sufficient sample collections. Utilizing this approach during experiment planning results in streamlined workflows with the integration of data across batches or experiments.

Within flow cytometry, defining a relevant research question with suitable parameters assists researchers with answering questions related to immunophenotyping, functional assays, and rare cell subset detection [96]. An example of a research question with low-dimensional data analysis may be a study evaluating the frequency and phenotypic features of CD4+ and CD8+ T cells in the SARS-CoV-2 mRNA vaccine for comparison between both groups [97]. However, a high-dimensional research question example may be an unbiased analysis of spectral flow cytometry analysis following vaccination for multiple cell population identifications, new phenotype combinations, and relative frequency determinations amongst groups [98]. For each designated data set, statistical methodologies should align with the research question and ensure validity, thereby allowing for meaningful interpretation of results that contribute to understanding the overall data set and the underlying mechanisms involved in the analysis.

**Figure 3:**
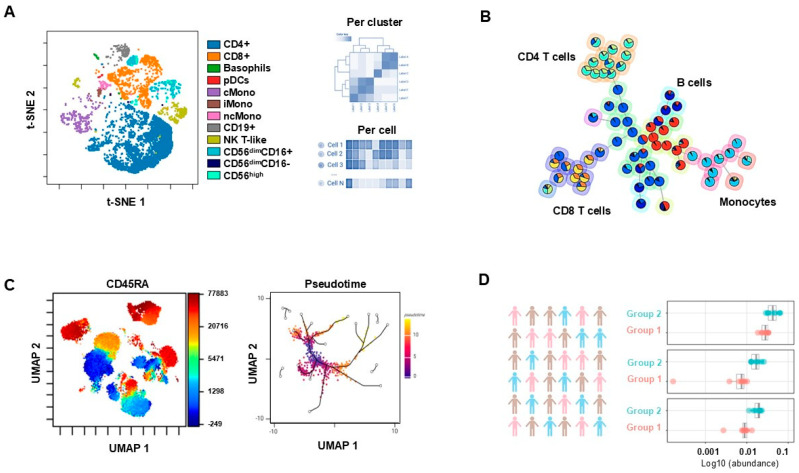
**Advanced single-cell data analysis.** (**A**) Automated identification of clusters of major cell populations based on fluorescence reported overlayed on a t-distributed stochastic neighbor embedding (t-SNE) map in a PBMC sample from a healthy donor. This enables the identification of populations as well as inter- and intra-comparisons. (**B**) Self-organizing map (SOM) resulting from a FlowSOM run on a PBMC sample from a healthy donor. The map identifies major clusters with colors indicating marker expression. (**C**) UMAP run with default settings on CD45+ cells in a PBMC sample from a healthy donor stained with a 40-color panel. Z channel for coloring CD45RA. Pseudotime representation enabling tracking of cell maturation/differentiation. (**D**) Results of a CITRUS run with a PAMR (nearest shrunken centroid model) used to identify clusters of cells with similar phenotypes that have a significantly different abundance between two groups of samples.

It is now possible to interrogate complex scientific/medical questions within clinical studies to address cell subset characteristics, apply cell population changes to clinical states, correlate target and reference cell populations, discern developmental branch point pathways, and detect changes in the biological state of cell populations [94]. These applications are specific to single-cell technologies such as flow cytometry and scRNA-seq (Figure 3). It is evident that current advances in computational biology have become significant for effective data analysis of the immunome. Single-cell sequencing has evolved beyond common transcriptomic profiling with scRNA-seq to generate diverse single-cell characterization [99,100]. Not only are applications of scRNA-seq helpful for understanding the immunological states, but they are also useful for providing cell biology comprehension as it relates to vaccination. This was elegantly demonstrated in patients with a special condition (*Lupus nephritis*) using machine-learning algorithms to develop predictive models for identifying key biological pathways, such as MTOR signaling, autophagy, toll-like receptors, and adaptive immunity to help identify potential mRNA vaccine developmental targets [101].

As conventional approaches assessing one individual marker are not sufficient for complex research questions, multiparametric analysis is becoming the norm or standardized approach. However, the determination of “gates” during flow cytometry data analysis is subjective and limited to a few dimensions, which prevents understanding of the full or total concept. Tackling the entire dimension of such complex data requires extensive experience and is also extremely time-consuming [102]. The use of automated analysis and dimensionality reduction algorithms such as t-distributed stochastic neighbor embedding (t-SNE) and uniform manifold approximation and projection (UMAP) can help to identify parameters with the largest variance, represent complex cytometric data with single two-dimensional plots, and improve data visualization (Figure 3). The analysis of separated clusters or individual cells within these clusters is then much easier to evaluate, thus providing information the two-dimensional data analysis cannot provide [103]. Clustering algorithms (Phenograph, FlowSOM) assist with combining cells into groups and allow cells to be used as separate groups within visualization plots to determine significant differences between treatments [104]. Defining pseudotimes as a proxy for continuous developmental trajectories could be an interesting avenue for analyzing the evolution of immune cell development/maturation. For example, it enables visualization of the developmental hematopoietic stem-cell trajectories into myeloid/lymphoid subsets [105].

Systems vaccinology is an integrative approach combining high-throughput technologies, computational biology, and immunology for comprehensive molecular and cellular analysis related to underlying vaccine response mechanisms. This field leverages multi-omics data, including genomics, transcriptomics, proteomics, and metabolomics, to create detailed immune response vaccination maps [106]. By applying systems biology approaches, researchers can identify early molecular signatures for vaccine efficacy predictions and unveil new protection resonates. This approach for signature blood transcription identification to vaccine-induced immunity has been described [107] and applied to specific vaccines such as malaria [108]. The combination of proteomic, metabolomic, and transcriptomic analyses to uncover biomarkers and immune pathways is linked to vaccine-induced protection, providing valuable insights for improving malaria vaccine design. It has also been applied to investigating the immune response to a novel HIV vaccine candidate. By integrating multi-omics metrics, researchers classified key immune cell populations and signaling pathways with strong and durable immune response correlations for highlighting potential vaccine efficacy targets [109]. These insights helped rationalize next-generation vaccine designs and personalized vaccination strategies aimed at enhancing immune protection across diverse populations.

Artificial intelligence (AI) and machine learning are two new tools used to facilitate the design of novel vaccines. Other impressive agents may also be used to accurately predict neoantigen structures for designing mRNA sequences to effectively target immune and cancer cells as well as optimize mRNA-lipid nanoparticle formulation for optimized delivery and stability [110]. This application is currently in use and has proven to be efficient for reducing the number of candidate targets and increasing the GO/NO-GO rate [111]. The use of AI can also help reduce the challenges (time, costs, epitope prediction, vaccine targeting, adjuvant selection, and vaccine coverage) that are inherent to vaccine design [112]. However, progress is warranted related to AI use for predicting immune response to vaccination [113]. Some studies have demonstrated the potential of supervised and unsupervised AI for understanding the response to treatment [114]. This was analyzed with a deep learning human cancer cell model that was trained on the responses of 1235 tumor cell lines to 684 drugs, suggesting that feeding the AI with significant amounts of immune response data to vaccine challenges would enable its use for prediction purposes. Such data required for these purposes may include responses of the immune system at multiple levels (molecular, organ, and organism) and diverse contexts (infection, oncology, cell biology, biochemistry, etc.) to provide a holistic representation of immune interactions.

The recent advances in technology described in this review enable the generation of immune system information with unprecedented depth for AI tool use as a combined source with other omics and clinical features to reveal new findings (cause-and-effect relationships between variables). These advancements could also be useful for testing the in silico effects of drug/vaccine perturbations on the immune response [115]. This entire tool represents a powerful solution for advancing knowledge on immune interactions, providing a framework to test and refine hypotheses about immune processes and facilitate translational applications. As there is a high heterogeneity and plasticity in the immune response among individuals, gathering multi-dimensional data on large cohorts may be needed to develop accurate predictive AI models and tools. The endeavors of the Human Cell Atlas project will greatly assist with accomplishing this goal.

## 7. Generating Robust and Reproducible Data with Flow Cytometry

**Figure 4:**
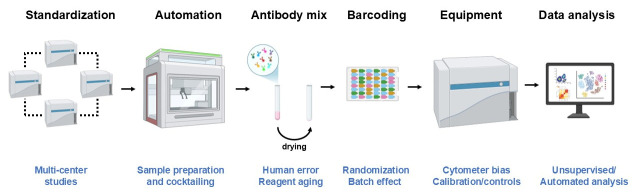
**Reducing the variability in flow cytometry analysis**. The standardization of cytometers enables comparisons of immunological data across various sites, as well as sites where different equipment is used for the same study. Automation can be integrated during various steps of experimental design, including sample preparation, cell stimulation, cell staining, and washing steps before analysis. The use of ready-to-use antibody mixes reduces the variability introduced by manual pipetting. Some ready-to-use mixes have a long shelf life (up to two years) with reduced logistical constraints. Another option is to introduce cell barcoding to the experimental design or to analyze samples from diverse study groups in the same batch. Ensuring the cytometer is well calibrated and controls are properly introduced will also reduce the variability and enable the normalization of data if required. Finally, automated analysis can be ideal for relieving the heavy burden as well as the subjectiveness of data analysis.

The quality of data generated with complex technologies depends highly on expertise and experimental design. The difficulty in replicating scientific data [116] applies to all fields, including immunology. Several biases could lead to reproducibility issues during experimentation. One such bias (statistical bias) is well known and pertains to the power of the study, reflecting the sample size of the population studied. Recently, data has suggested that an alternate bias (technical bias) is not well understood, although it is a strong predictor for reproducibility issues [117]. Examples of entities leading to replication challenges include equipment artifacts, reagents, and laboratory methods, as well as unstandardized protocols. Despite the immunology and cellular analysis challenges, there are ways to reduce variability and increase the generalization of findings from a study. One highly recommended suggestion includes focusing on techniques to increase the robustness of flow cytometry, which is the most widely used technology by immunologists (Figure 4).

In the context of multi-center and/or longitudinal studies, reducing the minimum variability introduced by human error (pipetting) is tremendously important. This principle was tested in a multi-centered study aimed at identifying immune signatures associated with kidney transplantation. The phase 1/2A clinical trial arising from The ONE Study included data from several countries (France, Germany, Italy, the UK, and the USA) with a 60-week follow-up after transplantation [118]. The group was able to perform standardized immune monitoring utilizing ready-to-use dried antibody mixes following the infusion of regulatory cell products. The results of the study revealed dysregulation in the myeloid (CD14^high^CD16^+^ monocytes) and lymphoid (CD8+CD28+ T cells, senescent T cells, and marginal zone-like B cells) compartments. To effectively evaluate infection, a similar technical approach was implemented to study the dynamics of human papillomavirus infection [119] and human monkeypox infection [120]. The same technique was also successfully applied in several SARS-CoV-2 vaccine studies [121]. For instance, the use of dried reagents was critical to ensure that exact amounts of antibody were used for CD169 mean fluorescence intensity (MFI) measurements, which reflected immune dysregulation, oxygenation need, and COVID-19 progression [122]. The use of antibody cocktails and the distribution of cryopreserved samples for several experimental groups across batches (randomization) may also decrease batch-related variability [123]. Furthermore, barcoding tends to be an ideal choice when fresh or cryopreserved samples are used to significantly minimize variability related to sample preparation and staining [124].

Adaptations to experimental design can be incorporated to reduce the variability introduced by human error (Figure 4) as well as the technical bias from standardized equipment. Many applications help to achieve this process for evaluating data generated from different cytometers [125]. However, the most challenging in flow cytometry involves comparing absolute numbers and the MFI of cellular markers generated from various cytometers. The IMI PRECISESADS study, a multi-centered research approach, used 11 different instruments to analyze fresh blood from 2559 individuals over a period of four years [126]. For standardization of all instruments, standard operating procedures (SOP) employed beads for inter-center harmonization and intra-center daily quality control to preserve intra-instrument stability, thus leading to the development of inter-instrument coefficients of variation (CVs) of less than 5%. The use of dried reagents enabled focusing on the equipment bias while minimizing sample preparation bias. During the interpretation steps, automated gating for population selection became applicable for reducing human error [127].

It is evident that many efficient tools exist to support automated gating when large numbers of samples require analysis and evaluation [128]. These tools also include ways to eliminate unwanted “events” that may arise from technical artifacts [129,130]. In this review, we have suggested a non-exhaustive list of solutions to increase rigor and reproducibility in flow cytometry experiment applications. Reducing the variability should enable an understanding of the real immune response following triggering with vaccination. This knowledge can only improve the quality and integration of data generated in clinical research and accelerate the development of vaccine candidates with optimal immune response and memory [131].

## 8. Conclusions

The analysis of the immune response to vaccination or natural infection is driving the development of the next generation of vaccines. The advent of extensive cellular analysis by flow cytometry and single-cell RNA sequencing has greatly contributed to the progress made in the past decades. Still, much more is needed in order to fully comprehend the responses to vaccination as they differ depending on the antigen, the mode of administration, the vaccine design, the health status, and the age of the recipient. To fully capitalize on the potential of single-cell technologies in vaccinology, ongoing efforts are essential to create more robust, cost-effective, and user-friendly platforms. Integrating single-cell data with other omics layers—such as proteomics, metabolomics, and epigenomics—could offer a comprehensive view of immune responses (Table 1). One aspect still to be developed in this context is the analysis of the cellular proteome and metabolic profile [132]. These techniques are currently being improved to increase throughput, as they often rely on imaging pairing [133,134]. As these technologies are utilized separately, the combination of these methods to generate data from a single cell would be ideal but is not possible in practice. While the profiling of gene expression, DNA methylation, and chromatin accessibility was successfully performed in the same cell [135], developing in silico models such as virtual twins (at the cellular level) may help combine information of the proteomic, genomic, lipidomic, and metabolic profiles. This may be possible with collaborative effort and the help of artificial intelligence [136]. In conclusion, single-cell analysis is a powerful tool for understanding the complexities of immune responses to vaccination. Although technical and logistical challenges persist, its full potential in research and clinical settings can be achieved through continued innovation and cross-disciplinary collaboration.

## Figures and Tables

**Table 1:** Comparison of technologies for single-cell analysis.

	Single-Cell Approach	Throughput	Experimental Workflow	Information Collected	Noise	Data Complexity	Cost/Expertise
**Flow cytometry**	Yes	High	Well-established	Scatter and fluorescence data	Moderate (photon and electronic noise)	Low	Moderate
**Spectral cytometry**	Yes	High	Requires advanced expertise in panel design	Scatter, fluorescence, and autofluorescence data	Moderate (photon and electronic noise)	Intermediate	Moderate
**Mass cytometry**	Yes	Moderate	Requires mastering chemistry of antibody conjugation	Time-of-flight data	Low (no photon)	Intermediate	Moderate
**RNA-seq/CITE-seq**	Adaptable to single-cell	High	Requires cell sorting/enrichment and advanced data analysis expertise	Gene-expression and surface markers expression data	Moderate (ambient RNA)	High	High
**ATAC-seq/WGSeq**	Adaptable to single-cell	Moderate	Requires cell sorting/enrichment and advanced data analysis expertise	Chromatin accessibility/DNA methylation	Moderate (dropout events)	High	High
**Proteomic**	Moderately adaptable to single-cell	Moderate	Standardized process to be developed	Protein profile by mass spectrometry	Moderate (electronic, shot, and chemical noises)	High	High
**Metabolomic**	Low adaptability to single-cell	Low (imaging-based)	Standardized process to be developed	Targeted/untargeted metabolic profile by mass spectrometry	Moderate (ion suppression, ion–ion interaction, and white noise)	High	High
